# Enhancing biological control of postharvest green mold in lemons: Synergistic efficacy of native yeasts with diverse mechanisms of action

**DOI:** 10.1371/journal.pone.0301584

**Published:** 2024-04-05

**Authors:** Martina María Pereyra, Mariana Andrea Díaz, Silvana Vero, Julián Rafael Dib

**Affiliations:** 1 Planta Piloto de Procesos Industriales Microbiológicos (PROIMI), Consejo Nacional de Investigaciones Científicas y Técnicas (CONICET), San Miguel de Tucumán, Tucumán, Argentina; 2 Área de Microbiología, Departamento de Biociencias, Facultad de Química, Universidad de la República, Montevideo, Uruguay; 3 Facultad de Bioquímica, Química y Farmacia, Instituto de Microbiología, Universidad Nacional de Tucumán, San Miguel de Tucumán, Tucumán, Argentina; University of Bari Aldo Moro, ITALY

## Abstract

Argentina is among the most important lemon fruit producers in the world. *Penicillium digitatum* is the primary lemon fungal phytopathogen, causing green mold during the postharvest. Several alternatives to the use of synthetic fungicides have been developed, being the use of biocontrol yeasts one of the most promising. Although many of the reports are based on the use of a single yeast species, it has been shown that the combination of agents with different mechanisms of action can increase control efficiency through synergistic effects. The combined use of native yeasts with different mechanisms of action had not been studied as a biological control strategy in lemons. In this work, the mechanisms of action of native yeasts (*Clavispora lusitaniae* AgL21, *Clavispora lusitaniae* AgL2 and *Clavispora lusitaniae* AcL2) with biocontrol activity against *P*. *digitatum* were evaluated. Isolate AgL21 was selected for its ability to form biofilm, colonize lemon wounds, and inhibit fungal spore germination. The compatibility of *C*. *lusitaniae* AgL21 with two killer yeasts of the species *Kazachstania exigua* (AcL4 and AcL8) was evaluated. *In vivo* assays were then carried out with the yeasts applied individually or mixed in equal cell concentrations. AgL21 alone was able to control green mold with 87.5% efficiency, while individual killer yeasts were significantly less efficient (43.3% and 38.3%, respectively). Inhibitory effects were increased when *C*. *lusitaniae* AgL21 and *K*. *exigua* strains were jointly applied. The most efficient treatment was the combination of AgL21 and AcL4, reaching 100% efficiency in wound protection. The combination of AgL21 with AcL8 was as well promising, with an efficiency of 97.5%. The combined application of native yeasts showed a synergistic effect considering that the multiple mechanisms of action involved could hinder the development of green mold in lemon more efficiently than using single yeasts. Therefore, this work demonstrates that the integration of native yeasts with diverse modes of action can provide new insights to formulate effective microbial consortia. This could lead to the development of tailor-made biofungicides, allowing control of postharvest fungal diseases in lemons while remaining competitive with traditionally used synthetic chemicals.

## Introduction

Citrus fruits are one of the main fruit crops worldwide. They are grown in more than 140 countries and exported around the world for their nutritional and therapeutic benefits [1]. Particularly, lemon fruit is one of the most important crops in Argentina in terms of production and export, and the main producing areas are in the province of Tucumán.

*Penicillium digitatum*, the causative agent of green mold in citrus, is responsible for serious economic losses during harvest, transportation and storage, thus limiting the commercial life of harvested fruit [2, 3]. The large amount of airborne spores not only represents an important source of inoculum in the field and during packaging, but can also cause allergic responses in humans [4]. Moreover, it has been recently proved that *P*. *digitatum* produces tremorgenic mycotoxins (tryptoquialanines A and C), capable of generating persistent or intermittent tremors in vertebrate animals [5].

For some years now, synthetic fungicides such as imazalil and thiabendazole have been the main means of controlling green mold decay [6]. However, the emergence of resistant fungal strains [7], their increasingly restricted use and the growing concern about contamination of food and the environment [8, 9] have promoted the search for alternatives and safer biological control strategies.

The use of yeasts with biocontrol activity targeting phytopathogenic fungi appears as a promising alternative to chemical pesticides [10–14]. Yeasts have been extensively studied as biocontrol agents due to their simple nutritional requirements adapting to the fruit’s environment, their ability to colonize surfaces for long periods [15, 16], their high tolerance to a wide range of temperatures, pH, and oxygen levels [17], their easy and rapid growth in bioreactors, and the greater acceptance by consumers as they are microorganisms commonly used in the food industry, either for the production of bread, wine and beer, or as nutritional supplement [18, 19].

While most research has focused on the individual application of biocontrol yeasts against green mold decay in citrus [20–26], less is known about their combined effect. Panebianco et al. [27] combined *Pseudomonas* strains with *Trichoderma* fungi to control *P*. *digitatum* in citrus; Agirman et al. [28] investigated the *in vitro* effect of combining *Aureobasidium pullulans* and *Meyerozyma guilliermondii* against *P*. *digitatum* in lemons; Öztekin et al. [29] combined three yeast strains (*Hanseniaspora uvarum*, *M*. *guilliermondii*, and *Metschnikowia pulcherrima*) for the biocontrol of *P*. *digitatum* in mandarins. To the best of our knowledge, there were no reports on the use of compatible antagonist yeast mixtures for the biological control of green mold decay in lemons.

The yeast *Clavispora lusitaniae* is commonly present in locally produced lemons [12, 30]. In previous studies, we investigated the antagonistic potential of different isolates of this species against green mold [12]. Besides lemon fruits, its notable action as a biocontrol agent was evident in sweet oranges, tangerines and grapefruits; however modes of action through which the yeasts exert their antagonistic activity are unknown. Furthermore, we have previously focused on native epiphytic lemon yeasts with killer phenotype as the main mechanism of action, showing promising results in the biocontrol of *P*. *digitatum* [30]. Therefore, the aim of this work was to study the mechanisms of action of *C*. *lusitaniae* isolates and determine the possible synergistic effect on the biocontrol of green mold in lemons by combining the most effective isolate with yeast exerting killer phenotype.

## Material and methods

### Microorganisms and fruit

*Penicillium digitatum* CSM/Pd-01 was kindly provided by the Phytopathology Lab of the citrus company San Miguel S.A. (Tucumán, Argentina). The pathogen was grown and maintained by regular subculturing on potato dextrose agar (PDA, 4 g/L potato extract, 20 g/L glucose, 15 g/L agar, Biokar, Allonne, France) at 25 ºC for 5 days. Spores were collected from plates in sterile saline solution containing 0.1% Tween 80 and adjusted to a final concentration of 10^6^ spores/mL, using a Neubauer chamber.

*Clavispora lusitaniae* AgL21 (MT649498.1), *C*. *lusitaniae* AgL2 (MT649496.1) and *C*. *lusitaniae* AcL2 (MT649495.1) were previously isolated from samples of a citrus packinghouse in Tucumán province [12]. They were grown and maintained on yeast extract peptone dextrose agar (YEPD agar, 5 g/L yeast extract, 10 g/L, peptone, 20 g/L glucose, 20 g/L agar, pH 4.5).

Eureka cultivar lemons (*Citrus limon* (L.) *Burm*) were harvested from local fields. These cultivars had not received any preharvest treatment with synthetic pesticides. Healthy fruits were transported to the laboratory for immediate use or storage at 8 °C for no more than 7 days. Selected fruits were free of any visible injuries or signs of rot and exhibited uniform size, shape, and ripeness.

### Modes of action

#### Biofilm formation

The ability of *C*. *lusitaniae* strains to form biofilm was determined using freshly squeezed lemon juice as the culture medium. Wells of a 96-well microtiter plate containing 180 μL of sterile juice were inoculated with 20 μL of the yeast suspension and incubated for 72 h at 25 °C. Negative controls containing only sterile lemon juice were included. After incubation, the wells were emptied, rinsed with water, and air-dried at room temperature. The adherent biofilm layer was stained with an aqueous solution of violet crystal 1% (w/v) for 20 min. The bound dye was eluted from each well with 200 μL of acetic acid and absorbance was measured at 620 nm.

Biofilm formation by yeasts in the presence of *P*. *digitatum* was also determined: 20 μL of the yeast was inoculated with 20 μL of the pathogen in a 96-well microtiter plates containing sterile lemon juice, and the same procedure described above was carried out. Biofilm formation was considered positive in those wells where the absorbance was greater than the mean of the negative control plus three standard deviations [31]. The assays were done in triplicate.

#### Siderophores production

Siderophore production was assessed by a modified Chromium Azurol Sulfonate (CAS) medium plate assay [32]. The culture medium was prepared without nutrients, serving as a revealing medium (overlay-CAS, O-CAS) [33]. The composition for 1 L of coating was: 60.5 mg CAS, 72.9 mg hexadecyltrimethylammonium bromide (HDTMA), 30.24 g piperazine-1,4-bis (2-ethanesulfonic acid) (PIPES), and 1 mM chloride ferric hexahydrate (FeCl_3_.6H_2_O) in 10 mL of 10 mM HCl. Agarose (0.9% w/v) was used as a gelling agent.

Each strain of *C*. *lusitaniae* was streaked onto YEPD agar plates and allowed to grow for 48 h at 25 °C. Ten mL of the O-CAS medium was poured on the grown microorganisms to determine the production of siderophores. After 30 min, the production of siderophores was observed by the color change in the superimposed medium that exclusively surrounds the producing microorganisms: from blue to purple (for catechol-type siderophores) or from blue to orange (for microorganisms that produce hydroxamates). As a positive control, a strain of *Leucosporidium scottii* At17 capable of producing siderophores was used [34].

#### Wound site colonization

The ability of *C*. *lusitaniae* AgL21 to colonize lemon wounds was evaluated using the technique described by Perez et al. [15] with some modifications. Five lemons were selected, disinfected with 70% ethanol, and wounded once in the equatorial zone (2 mm deep x 1 mm wide). They were subsequently dipped in a yeast suspension (10^8^ cells/mL) for 2 min, placed in plastic trays and incubated at 25 °C. To monitor yeast growth in the wound, tissue samples containing the entire wound were taken (5 mm in diameter) at days 0, 1, 3, 5 and 7 of incubation, and subsequently placed in microtubes with 1 mL of saline solution added with 0.1% Tween 80. Samples were shaken for 20 min at 180 rpm. Dilutions of each sample were made and seeded on YEPD agar plates supplemented with ampicillin (100 μg/mL) and chloramphenicol (50 μg/mL). Yeast colonies were counted after 48 h and a growth curve was constructed by plotting the logarithm of viable cells in each wound versus storage time.

Wound colonization capacity of *C*. *lusitaniae* AgL21 at low temperature was also evaluated. For this, treated lemons were incubated at 8 °C and wound samples were taken at 10, 20, 30 and 40 days. Both assays were performed in triplicate.

#### Scanning electron microscopy of inoculated wounds

The effect of wound colonization by yeast strain AgL21 on the development of *P*. *digitatum* was visualized by scanning electron microscopy (SEM). Lemons were disinfected, wounded and dipped in the yeast suspension as described above, and 24 h later they were dipped in a *P*. *digitatum* spore suspension (10^6^ spores/mL). After 72 h of incubation, tissue samples containing the entire wound were taken. Samples were fixed by immersion in Karnovsky’s fixative (pH 7.2 phosphate buffer, 1.7% glutaraldehyde, 2.7% paraformaldehyde) for 24 h [35]. After that, they were dehydrated in a series of ethanol concentrations and passed through 100% acetone twice before the critical point of drying with CO_2_ (Denton Vacuum). Dried tissues were mounted on gold-coated aluminum chips (JEOL Ion Sputter JFC 1100, USA) and observed using a Zeiss SUPRA 55 VP scanning electron microscope (SEM (Oberkochen, Germany).

#### Effect on pathogen spore germination

The assay was performed in a 96-well microtiter plates containing 100 μL of potato dextrose broth (PDB) medium inoculated with 10 μL of a suspension of *P*. *digitatum* (10^6^ spores/mL) and 10 μL of a suspension of *C*. *lusitaniae* AgL21 (10^8^ cells/mL). Wells containing pathogen spores without the addition of AgL21 cells served as sporulation controls. Additionally, a strain of *Candida catenulata* M1.4 was used as a negative control of sporulation inhibition [15]. The microplate was incubated for 12 h at 25 °C. Spore germination rate was recorded by viewing at least 100 spores at a time. The treatment consisted of three replications and the experiment was repeated twice.

#### Production of antifungal enzymes

Production of chitinase and β-1,3-glucanase by *C*. *lusitaniae* AgL1 was assayed in the presence of fungal cell wall (FCW) of *P*. *digitatum*, as described Vero et al. [36], with modifications. The fungus was grown in yeast extract sucrose medium (YES, 5 g/L yeast extract and 15 g/L sucrose) at 25 °C for 5 days. Obtained mycelia was dried with sterile filter paper and crushed in a sterile mortar in the presence of liquid nitrogen until a fine powder was obtained; it was suspended in 5 M NaCl, sonicated for 5 min and centrifuged at 7,000 rpm (TGL16 Table Top High Speed Centrifuge, Yingtai, Huntan, China) for 20 min. The supernatant was discarded, and the precipitate was washed three times with distilled water. FCW was dried in Petri dishes at 60 °C for 3 h and the obtained powder was added to yeast nitrogen base medium (YNB) at a concentration of 1 mg/mL and autoclaved. The resulting medium was inoculated with AgL21 strain (10^8^ cells/mL) and incubated at 25 °C for 7 days. Additionally, YNB medium without FCW was also inoculated. Aliquots of the cultures were filtered daily through a 0.45 mm nitrocellulose filter.

Chitinase activity was assayed as described by Mahadevan et al. [37], measuring the release of p-nitrophenol from p-nitrophenyl N-acetylglucosamidine [pNP(GlcNAc)]. pNP(GlcNAc) (Sigma-Aldrich, EEUU) was dissolved in 0.05 M potassium acetate buffer (pH 6). In a microplate, 10 μL of the filtered culture was added to 90 μL of the 0.18 mmol/L p-nitrophenyl reagent, and incubated at 37 °C for 6 h. The reaction was stopped by adding 10 μL of 1 mol/L NaOH. Absorbance was measured at 405 nm in a microplate reader (Bio-tek Instruments, Winooski, VT, USA). One unit of enzyme was defined as the amount that releases 1 mmol of p-nitrophenol per mg of protein.

β-1,3-glucanase production was tested as in Masih el at. [38]. A reaction mixture was prepared by adding 62.5 μL of 0.05 M potassium acetate buffer (pH 6) containing 1% laminarin to 62.5 μL of filtered culture and incubated at 37 °C for 6 h. Glucanase activity was determined as the amount of reducing sugars released from laminarin, measured according to the Nelson-Somogyi method [39, 40] using glucose as a standard. One unit of β-1,3-glucanase was defined as the amount of enzyme that liberates 1 mmol of reducing sugars per mg of protein.

#### *In vitro* determination of yeasts killer phenotype

**Molecular identification**. Killer activity determination was carried out on 43 yeasts isolated in our previous work, with proven antagonistic activity against *P*. *digitatum* [12].

The killer assay was performed using the plate diffusion technique described by Perez et al. [30]. *Saccharomyces cerevisiae* CEN.PK2-1c [41] was used as the reference sensitive strain, while *Kazachstania exigua* 120 [30] and *S*. *cerevisiae* GS1731 [42] served as positive and negative controls for killer activity, respectively. The assay was conducted in Petri dishes with YEPD agar medium (pH 4.5) added with 0.003% (w/v) methylene blue and 2% NaCl [43]. Killer activity was evidenced by a clear zone of inhibition around the colonies.

DNA extraction from those isolates that tested positive for killer activity was performed as described in Pereyra et al. [12]. Taxonomic identification was achieved by PCR amplification of the D1/D2 domain of the 26S rRNA gene using primers NL-1 (5′-GCA TAT CAA TAA GCG GAG GAA AAG-3′) and NL-4 (5′-GGT CCG TGT TTC AAG ACG G-3′) [44]. Sequencing of the purified PCR products was performed at Microsynth Seqlab (Göttingen, Germany). The obtained sequences were processed using Clone Manager 9 Software (Cary, NC, USA), and sequence similarity searches were performed with the BLAST network service of the NCBI database (http://www.ncbi.nlm.nih.gov/BLAST). The sequences of these killer isolates have been deposited in the GenBank database under the following accession numbers: OL780468 (AcL4) and OL780469 (AcL8).

### Combination of biocontrol yeasts

#### *In vitro* compatibility of yeasts

In order to determine if the protection efficiency of *C*. *lusitaniae* AgL21 could increase in combination with yeasts strains showing killer phenotype, their compatibilities were first determined. 200 μL of *C*. *lusitaniae* AgL21 (10^8^ cells/mL) were mixed with melted YEPD agar and was poured into a Petri dish; once solidified, killer yeasts were spotted on the agar surface. The plate was incubated at 25 °C for 48 h and it was determined whether the yeasts were compatible by evaluating the presence or absence of an inhibition halo around the killer colonies.

#### *In vivo* biocontrol assay against *P*. *digitatum*

The effect of the combination between the yeasts was carried out by individually combining yeast AgL21 with compatible killer yeasts (AcL4 and AcL8). A total of 60 lemons per yeast were used (4 replicates of 15 lemons). Lemons were disinfected, wounded once in the equatorial zone with an awl (2 mm deep × 1 mm wide), placed in mesh bags and dipped for 2 min in yeast suspensions. Yeast cultures (*C*. *lusitaniae* AgL21, AcL4 isolate and AcL8 isolate) and their mixtures [M1 = AgL21+AcL4 and M2 = AgL21+AcL8 (1:1, v/v; 10^8^ cells/mL)] were prepared by combining equal volumes of suspensions of each antagonist. The treated fruit was incubated at 25 °C for 24 h and then dipped in the *P*. *digitatum* suspension (10^6^ spores/mL) for 2 min. Yeast protection efficiency (PE) was calculated after 5 days of incubation using the formula: protection efficiency (%) = (number of healthy fruit /numbers of total fruits) × 100.

### Statistical analysis

All data obtained from the *in vivo* experiments were subjected to analysis of variance according to parametric and non-parametric approaches (InfoStat/L software, Córdoba, Argentina) [45]. All percentage data were transformed using arcsine (sin-1 square root x) prior to statistical analysis. Data were analyzed using ANOVA and mean values were compared with Tukey’s test at the 5% significance level.

Limpel’s formula, described by Richer et al. [46], was used to assess synergism. The formula is: Ee = X + Y—(XY/100), where Ee represents the expected effect from the additive response of two treatments, and X and Y represent the percentage protection efficiencies observed with each agent used alone. Therefore, if the combined protection efficiency of the two agents exceeds Ee, synergism is considered to be present.

## Results and discussion

### Modes of action

#### Biofilm formation and siderophores production

Microorganisms compete with each other and with their hosts for nutrients and space; which is considered the main mode of action among several biological control yeasts [47, 48]. Competition for space and nutrients is effective when the antagonist is present in sufficient number at the right time and place, and is able to use limited resources more efficiently than the pathogen [14]. Once antagonistic yeasts come into contact with the wounded fruit surface, they colonize the wounds and rapidly deplete nutrients, limiting fungal spore germination. Afterward, further action mechanisms—inhibition of spore germination, production of antifungal enzymes—will be involved to cooperatively control postharvest pathogens [49, 50].

Biofilm formation can be considered a specific and highly successful strategy to compete for space with the pathogen. In this process, microorganisms form multicellular structures embedded in a complex matrix that allows them to improve their adhesion to surfaces and resistance to stress. It is considered an important attribute of postharvest antagonists, aiding their ability to successfully colonize and protect both wounds and the fruit surface [50, 51]. In this regard, the ability of *C*. *lusitaniae* AgL21, AgL2 and AcL2 to form biofilms was determined in a microplate assay using lemon juice as a culture medium, in the presence and absence of *P*. *digitatum*. Table 1 shows the results represented by the absorbance measurements. In both treatments, the control absorbance (lemon juice) yielded similar OD values: 0.245 in the absence of the pathogen and 0.225 in its presence; this would indicate that the fungus is not capable of producing biofilm on its own and, therefore, its presence would not interfere with the OD quantification. Without *P*. *digitatum*, the only yeast capable of forming biofilm was *C*. *lusitaniae* AgL21, taking as reference the OD limit value for biofilm formation of 0.386 (control absorbance plus three standard deviations). When the pathogen was included (OD limit = 0.279), the three strains were capable of forming biofilm; however, AgL21 differed statistically from the rest. Although biofilm formation by *C*. *lusitaniae* AgL21 was higher in the presence of the fungus, no significant difference was observed with respect to its behavior without the fungus.

**Table 1:** Biofilm formation by *C*. *lusitaniae* strains in the presence and absence of *P*. *digitatum*.

Treatment	A_620_ (media ± standard deviation)	
	Without *P*. *digitatum*	With *P*. *digitatum*
Control*	0.245 ± 0.047^a^	0.225 ± 0.018^a^
*C*. *lusitaniae* AgL21	0.476 ± 0.052^b^A	0.645 ± 0.165^b^A
*C*. *lusitaniae* AcL2	0.303 ± 0.015^a^	0.419 ±0.141^ab^
*C*. *lusitaniae* AgL2	0.287 ± 0.072^a^	0.445 ± 0.004^ab^

*Squeezed lemon juice. Treatments with the same letter in the same column are not significantly different (p < 0.05). Same capital letters in the same row do not differ significantly from each other (p < 0.05).

The molecular underpinnings of the process and the composition of different biofilms have only been studied in detail for *Pichia fermentans*, capable of producing biofilm on apple wounds to protect them against postharvest diseases [52]. Biofilm formation has also been reported to be a key mechanism of action involved in the protective and biocontrol activities of *Aureobasidium pullulans* [22, 53], *Kloeckera apiculate* [54], *Pichia kudriavzevii* [55], *Wickerhamomyces anomalus* [56] and *Metschnikowia pulcherrima* [57]. To our knowledge, this is the first report concerning biofilm formation in *C*. *lusitaniae* species as fruit wound colonizing agents.

**Fig 1:**
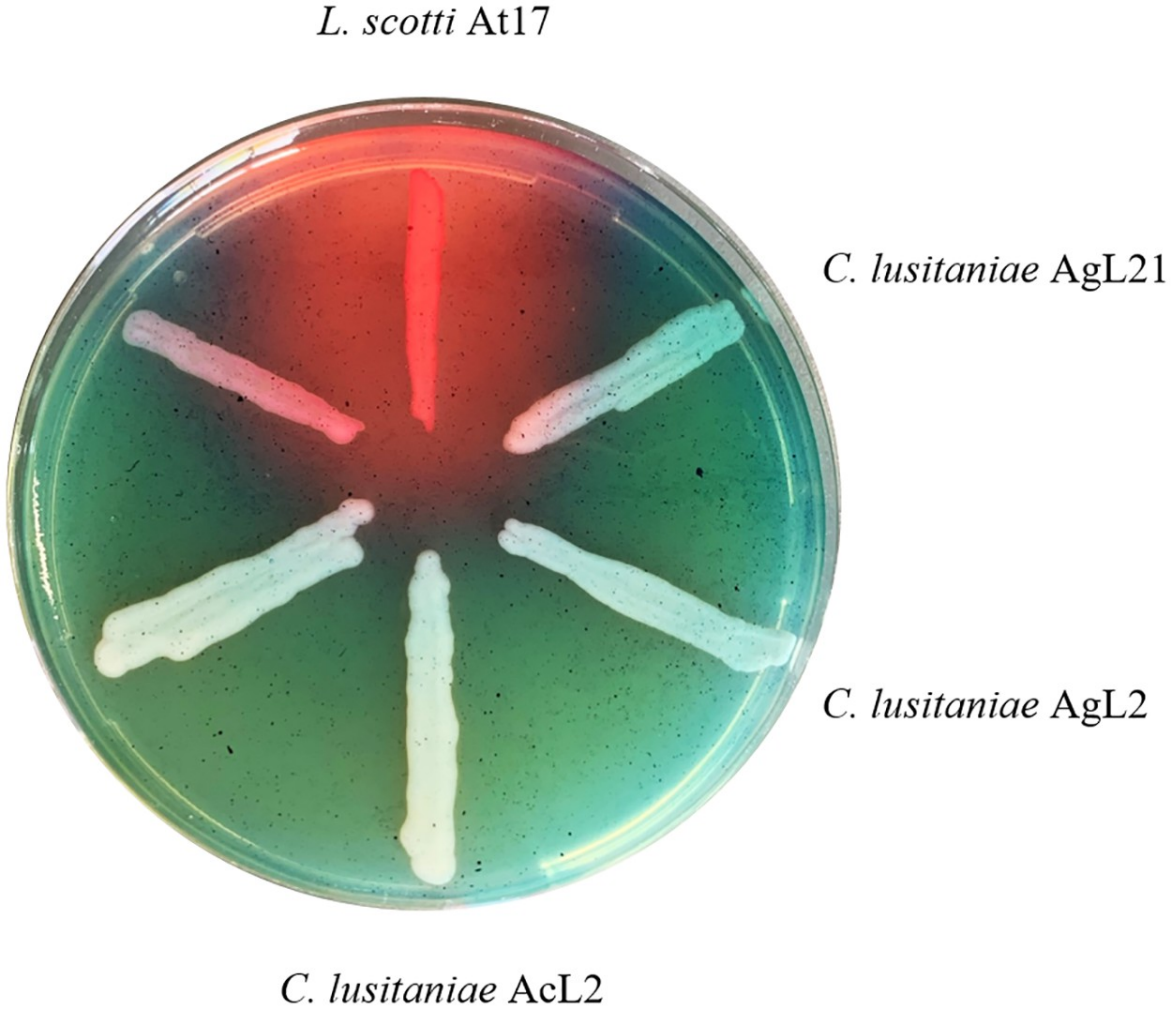
Siderophores production on plates with O-CAS medium. Orange halo around the streak indicate siderophores production. A strain of *L*. *scotti* At17 was used as a positive control.

Regarding nutrients, the fruit wound is a low-oxygen, low-iron microenvironment, thus yeasts could benefit by producing siderophores to compete for iron and interfere with pathogen germination, growth, and virulence [14]. Siderophores production by yeast strains was evaluated using the O-CAS medium. It was observed that none of the yeasts was capable of producing these iron chelating agents, neither of the hydroxamate type nor of the catechol type, in comparison with *L*. *scotti* At17 used in this assay as a positive control (Fig 1). The lack of competition for iron by yeasts suggests that other mechanisms are involved in their biocontrol activity. Nitrogen, another key nutrient limiting postharvest pathogen growth, is also worth investigating due to the abundance of sugars and scarcity of nitrogen sources in most fruits [58]. Further studies are needed to confirm the significance of nitrogen sources in the biocontrol efficacy of these yeasts.

#### Wound site colonization

**Fig 2:**
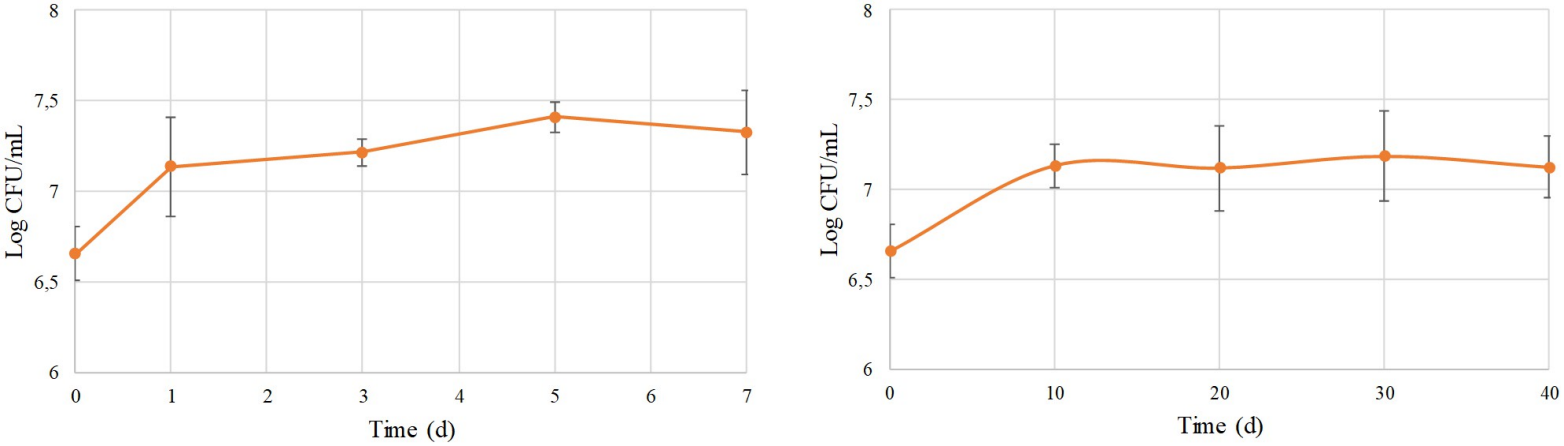
Population dynamics of *C*. *lusitaniae* AgL21 in lemon wounds at 25 °C (A) and 8 °C (B). The error bars indicate the standard deviations.

Based on the biofilm formation results, the ability of *C*. *lusitaniae* AgL21 to colonize wounds during 7 (25 °C) or 40 (8 °C) days of incubation was determined. Both at room (Fig 2A) and cold temperatures (Fig 2B), yeast development was similar: the yeast grew and remained viable throughout the whole incubation time. At 25 ºC, AgL21 grew exponentially during the first day of incubation; then the growth rate remained stable, reaching a population density of 7.30 log CFU/mL. At 8 ºC, the yeast growth was higher in the first 10 days of incubation, reaching a final population of 7.12 log CFU/mL at 40 days. Probably, such ability to colonize wounds and compete with the pathogen for space is the determining factor why these *C*. *lusitaniae* isolates have been efficient in the control of green mold when they were evaluated in different citrus species [12]. According to Liu et al. [11], among the biocontrol agents, yeasts turn out to be the ones that mainly compete for space and some nutrients with the pathogens, being able to grow rapidly during the first 24 h after treatment, depleting available nutrients and physically occupying the wounds. Generally, they exert a fungistatic effect on the pathogen, resulting in an inhibition of spore germination, which remains alive and capable of germinating after the addition of other nutrients [59]. For this reason, from the first day after treatment, other modes of action could participate and be decisive for the success of disease control [51]. Numerous reports have demonstrated the ability of different yeast species to colonize wounds and prevent the development of postharvest pathogens [15, 21, 34, 60, 61].

#### Scanning electron microscopy

**Fig 3:**
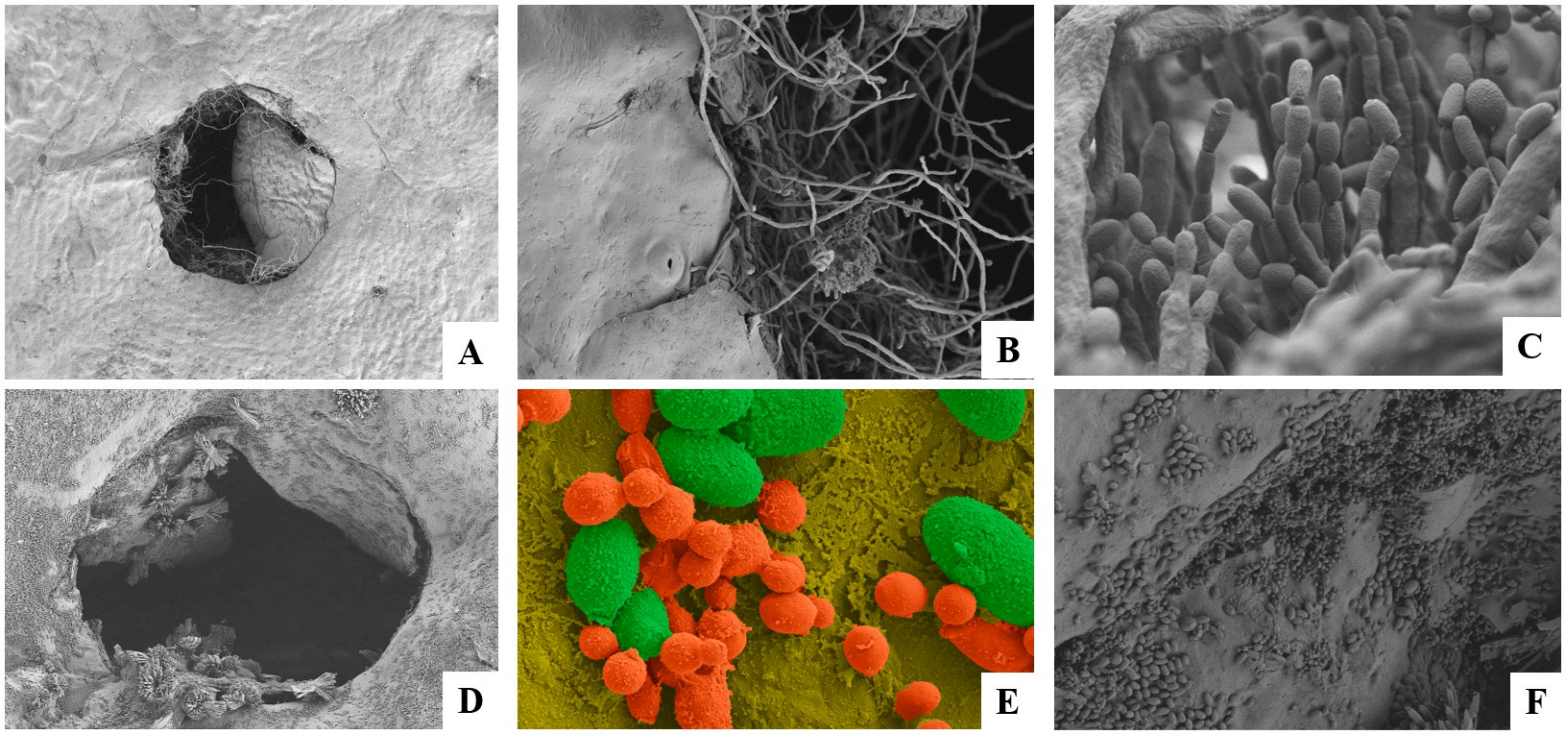
SEM of lemon wounds after 3 days of treatment with *C*. *lusitaniae* AgL21 and *P*. *digitatum*. **(A) (B)** and **(C)** show the lemon wound infected only with the pathogen. **(D) (E)** and **(F)** represent the wound previously treated with the yeast. Image F was digitally colored with Adobe Photoshop CS6: *P*. *digitatum* spores are represented in green; cells of *C*. *lusitaniae* AgL21 are represented in orange. The magnifications were as follows: 100X (A), 1.00 KX (B), 5.00 KX (C, F), 69X (D), 500X (E).

Wound colonization by *C*. *lusitaniae* AgL21 and its effect on the mycelial development of *P*. *digitatum* was observed by SEM. As reported by Janisiewicz et al. [59], yeasts can exert a fungistatic effect on the pathogen, resulting in an inhibition of spore germination. This could be verified under electron microscopy observations. It was determined that those lemons infected only with *P*. *digitatum* showed a high level of spore germination and mycelial growth on the third day of incubation (Fig 3A–3C), while wounds protected by the yeast revealed the ability of AgL21 strain to colonize and inhibit the pathogen conidia germination. Non-germinated *P*. *digitatum* conidia could be observed both inside the lemon wound (Fig 3E) and on the surface (Fig 3F). In addition, there was no evidence of fungus mycelial development. This mechanism has been reported for several biocontrol yeasts, including those that inhibit mycelial growth [15, 25, 62] and those that do not [36]. Having proven in previous studies the ability of *C*. *lusitaniae* AgL21 to produce soluble [12] and volatile organic compounds with fungistatic or fungicidal activity against *P*. *digitatum* [23], it could be hypothesized that the sum of all such mechanisms could restrict spore germination.

#### Effect on pathogen spore germination

**Fig 4:**
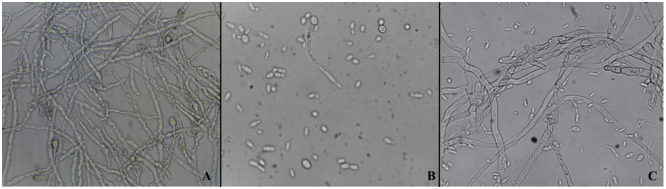
Effect on the inhibition of spore germination of *P*. *digitatum* after 12 h of incubation. **(A)** Germinated spores of untreated *P*. *digitatum*. **(B)** *P*. *digitatum* in the presence of *C*. *lusitaniae* AgL21 cells. **(C)** *P*. *digitatum* in the presence of *C*. *catenulata* M 1.4 cells, yeast unable to inhibit spore germination of the pathogen.

The assay was performed in a 96-well plate using PDB medium inoculated with a suspension of *P*. *digitatum* and a suspension of *C*. *lusitaniae* AgL21. Results showed that the presence of AgL21 significantly inhibited spore germination of the pathogen (Fig 4). A 62% inhibition was achieved in relation to the 11% produced by the species *C*. *catenulata* M 1.4, which did not differ significantly from the control (S1 Table). These results are in agreement with the observations made under the electron microscope.

#### Production of antifungal enzymes

The secretion of enzymes such as chitinases [63, 64], glucanases [36, 65] or proteases [66, 67] is commonly reported and highlighted in antagonistic yeasts as a potential cause of their biocontrol activity, as they facilitate the breakdown of cell wall components in plant pathogens, leading to cell lysis and leakage of cytoplasmic nutrients. Here, the production of fungal cell wall-degrading enzymes by *C*. *lusitaniae* AgL21 could not be detected even when grown in the presence of *P*. *digitatum* cell wall as the sole carbon source detected. This suggests that the greatest potential of AgL21 as a biocontrol agent is based mainly on its ability to colonize the fruit wound through the formation of a microbial biofilm and inhibit the germination of fungal spores by competing with the pathogen for space and nutrients.

#### *In vitro* determination of yeasts killer phenotype

**Fig 5:**
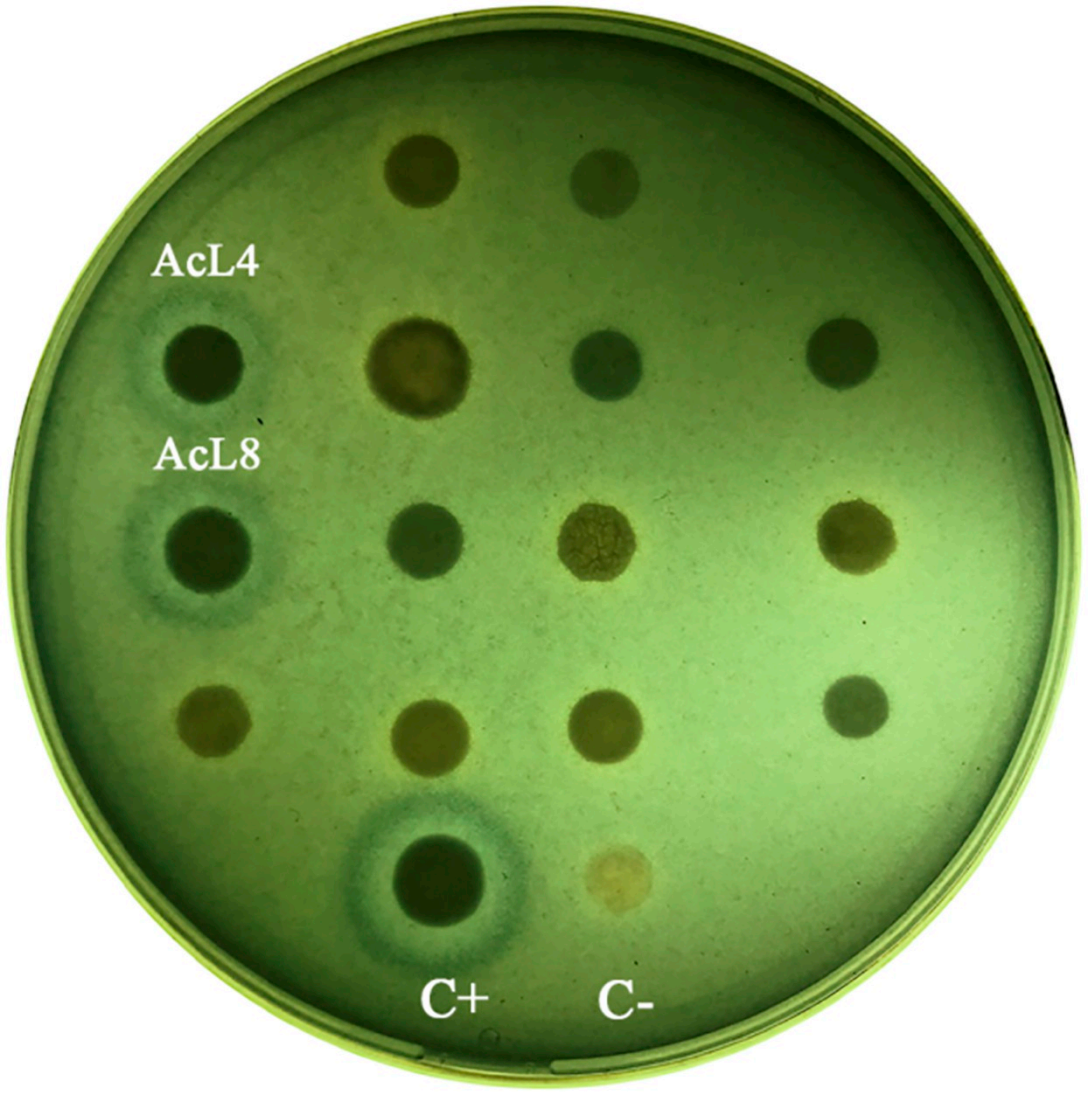
Plaque diffusion assay in YEPD medium to determine the killer phenotype of the isolated yeasts. C+ (positive control) corresponding to the yeast *K*. *exigua* 120 and C- (negative control) corresponding to the strain *S*. *cerevisiae* GS1731.

**Yeast molecular identification**. The presence of the killer phenotype was determined for the 43 yeasts that were able to inhibit the development of *P*. *digitatum* in our previous work. Only two yeast strains, AcL4 and AcL8 depicted a killer phenotype (Fig 5). The yeasts AcL4 (OL780468) and AcL8 (OL780469) were identified as belonging to the genus *Kazachstania exigua*, showing 100% identity with the reference strain *K*. *exigua* LC219508.1. In previous isolations, Perez et al. [30] described a killer phenotype for this species. The strain *K*. *exigua* 120 was found to inhibit the *in vitro* development of *P*. *digitatum*, *Penicillium italicum* and *Phomopsis citri*, as well as the development of human bacterial pathogens.

### Combining biocontrol yeasts

There is a growing interest among researchers in the use of combinations of antagonistic microorganisms to exploit the possible synergistic effects and to improve the control strategies [29]. Since the combination of biocontrol yeasts with different mechanisms of action can ideally result in more efficient protection, the combined application of *C*. *lusitaniae* AgL21 with killer yeasts was evaluated against *P*. *digitatum* in lemon. Combinations of non-competitive yeasts can have a broader range of activity and be more effective in the treatment of postharvest diseases [27, 68]. For this reason, the compatibility of AgL21 strain with *K*. *exigua* AcL4 and AcL8 was determined in a Petri plate assay. As a result, no growth inhibition of AgL21 was observed around the killer colonies.

**Fig 6:**
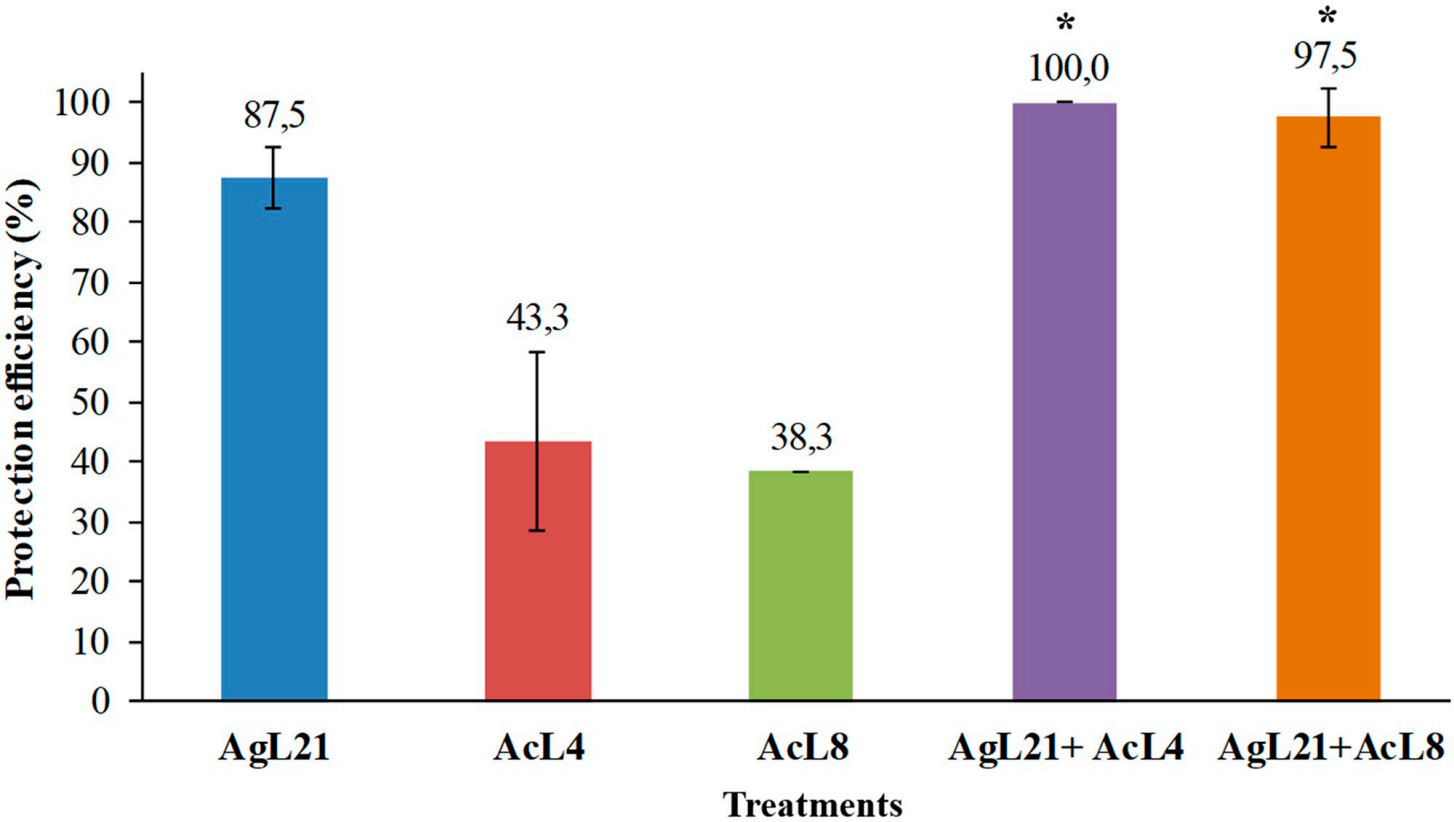
Protection efficiency of *C*. *lusitaniae* AgL21 alone and in combination with *K*. *exigua* AcL4 and AcL8. The error bars indicate the standard deviations. Identical letters are not, according to Tukey’s test, significantly different (p > 0.05). Asterisk indicates synergistic activity was present according to Limpel’s formula.

Yeasts combinations were then evaluated against *P*. *digitatum* in lemon. Protection efficiencies of the individually tested killer strains were rather low: *K*. *exigua* AcL4 reached 43.3%, while strain AcL8 only achieved 38.3%. *C*. *lusitaniae* AgL21 protected lemon wounds with 87.5% efficiency. When combined yeasts were applied, the protection efficiency increased up to 100% and 97.5%, for M1 and M2 treatments, respectively (Fig 6). These results showed a synergistic effect in the use ofAgL21 strain together with the killer yeasts *K*. *exigua* AcL4 or AcL8, compared to individual treatments.

There are several genera of yeast capable of producing killer proteins. These toxins are predominantly active at acidic pH and have lethal activity against sensitive microorganisms [69]. On citrus surface, the pH is less acidic than inside, and drops sharply when the fruit is wounded or injured, which reduces the effectiveness of conventional fungicides. The incorporation of killer toxin-producing biocontrol yeasts could be a promising and effective approach to protect citrus and fruits in general. Some authors such as Platania et al. [26], for example, reported that different killer species such as *W*. *anomalus* and *S*. *cerevisiae* were able to control the green mold disease in Tarocco oranges. Da Cunha et al. [70] also found strains of *Candida stellimalicola* and *S*. *cerevisiae* capable of antagonizing *P*. *italicum* in “Valencia” sweet oranges. In addition to citrus fruits, the use of killer yeasts as postharvest biocontrol agents was successfully reported in apple [71], peach and plum [72], tomato [73], and papaya [74].

In most of the studies related to the biological control of plant pathogens, only a single biocontrol agent is applied [36, 75–77]. However, attempts have been made to apply more than one. Guetsky [78] suggested the application of *Pichia guilliermondii* and *Bacillus mycoides* to prevent *Botrytis cinerea* infection in strawberry leaves. In other studies, simultaneous application of more than two biological control agents was attempted to improve biocontrol efficacy; for example, combinations with three microbial antagonists (yeast, bacteria and fungus) in grapes significantly reduced *B*. *cinerea* infection [79]. As the use of mixtures of microorganisms is an emerging approach in the field of postharvest diseases biocontrol, we suggest that the application of more than one antagonist will increase reliability and decrease variability of biological control.

## Conclusion

Developing successful commercial formulations and effective application strategies requires a thorough understanding of the individual mechanisms of action employed by biocontrol agents. The use of different yeasts strains with diverse modes of action holds promise for enhancing the effectiveness of postharvest rot control in lemons by expanding the biological control potential of these agents. Our findings demonstrated a synergistic effect between compatible yeasts, as evidenced by the significantly increased biocontrol activity against *P*. *digitatum* when *C*. *lusitaniae* AgL21 was combined with killer yeasts *K*. *exigua* AcL4 or AcL8. This highlights the potential of integrating antagonistic yeasts with diverse mechanisms of action as a novel approach towards formulating effective microbial consortia that can serve as tailor-made biofungicides for the biological control of postharvest fruit diseases.

## Supporting information

S1 Table Influence of *C*. *lusitaniae* AgL21 on *P*. *digitatum* spore germination. (DOCX)
