# Supplementary material for: Enhancing biological control of postharvest green mold in lemons: Synergistic efficacy of native yeasts with diverse mechanisms of action
Source: PLoS One. 2024 Apr 5;19(4):e0301584. doi: 10.1371/journal.pone.0301584 (PMC10997081; doi:10.1371/journal.pone.0301584)
Supplement: S1 Table — (DOCX) [file pone.0301584.s001.docx]

**S1 Table. Influence of *C. lusitaniae* AgL21 on *P. digitatum* spore germination.**

| Treatment | *P. digitatum* spores germination (%) |
| --- | --- |
| Control | 94^a^ |
| *C. lusitaniae* AgL21 | 38^b^ |
| *Candida* *catenulata* M 1.4 | 89^a^ |

The control represents spore germination in the absence of AgL21 strain. Each value is the mean of three experiments. Values with the same letter are not significantly different (p > 0.05).
